# Molecular Mechanisms of Metal Toxicity and Plant Tolerance

**DOI:** 10.3390/ijms24097810

**Published:** 2023-04-25

**Authors:** Michael Moustakas

**Affiliations:** Department of Botany, Aristotle University of Thessaloniki, GR-54124 Thessaloniki, Greece; moustak@bio.auth.gr

Increased industrial and agricultural human activities, such as mining and smelting, electroplating, wastewater irrigation, and chemical fertilizers, have resulted in high environmental concentrations of toxic metals [1]. It is now well-recognized that the increased concentrations of some non-essential metals to living organisms, such as cadmium (Cd), lead (Pb), nickel (Ni), arsenic (As), or chromium (Cr), accumulate in the environment and subsequently become toxic to all organisms [1,2,3]. Environmental pollution by toxic heavy metals is increasing worldwide and poses an increasing hazard to the environment and human health [1,2,3]. Chromium (Cr) exists in soil in several oxidation states, from −2 to +6, and in various complex ions; the hexavalent Cr compounds [Cr(VI)] have proven to be carcinogenic for humans [3]. Soil contamination with Cr(VI) considerably reduces the genetic diversity and richness of bacteria and fungi, and significantly disturbs the growth and development of maize plants [3]. However, humic acids totally counteract the effects of Cr toxicity on *Zea mays* plants [3].

Under normal, natural conditions, cadmium (Cd), a non-essential element in plants, exists in low concentrations, but with the development of modern industry, Cd has been widely applied in many fields as a semiconductor, in electroplating, and as feed and fertilizer, resulting in the pollution of the atmosphere and the water [2]. The use of livestock manure and mineral fertilizers increased the accumulation of Cd in farmland and, due to its high mobility and toxicity, has become increasingly serious for humans through the food chain [2]. The recent review by Chen et al. [2] summarizes the effects of Cd on rice growth, yield, and quality; the physiological and molecular mechanisms of Cd absorption in the roots; the loading and transport of Cd in the xylem; the distribution of Cd in nodes; the redistribution of Cd in leaves; the accumulation of Cd in the grains; and the regulation mechanism of the Cd stress response. In addition, the genetic regulation mechanism of Cd accumulation in rice is reviewed, as well as the strategies to reduce Cd content in rice for the safe use of Cd-contaminated soils [2].

However, even essential micronutrients for humans, animals, and plants, such as copper (Cu), that participate in various morphological, physiological, and biochemical processes can be toxic to plant growth and development [4]. Copper plays an important role in photosynthesis, respiration, the antioxidant system, and signal transduction [4]. Nevertheless, numerous studies have revealed the adverse effects of excess Cu on crop germination, plant growth and photosynthesis, and the antioxidant activities [4]. The biological functions of Cu, the toxicity of excess Cu, the process of Cu transportation in plants and the roles of Cu’s transportation of proteins and chaperone proteins, as well as the mechanisms of the detoxification and tolerance of Cu in plants, are summarized in another review by Chen et al. [4].

Plants growing on metal-polluted sites tolerate high metal concentrations by restricting the metal’s transportation to the aerial tissues [1,5]. However, some plant species can accumulate metals at very high concentrations in their aerial parts and are called hyperaccumulators [1,5,6]. For example, foliar Cd concentrations above 100 μg g^−1^ dry biomass (0.01%) are considered extraordinary and a threshold value for Cd hyperaccumulation [1,5,6]. For zinc (Zn), leaf concentrations above 300 μg Zn^2+^ g^−1^ DW (0.03% dry mass) are considered toxic to plants [5]. However, plant species that are extremely tolerant to Zn can hyperaccumulate more than 10,000 μg Zn^2+^ g^−1^ DW without displaying toxicity symptoms [5]. The exposure of *Salvia sclarea* plants to excess Zn resulted in increased Ca, Fe, Mn, and Zn concentrations, but decreased Mg, in the aboveground tissues [5]. The decreased Mg content in the leaves contributed to a decreased chlorophyll content that reduced the excess absorption of sunlight and improved photosystem II (PSII) photochemistry (Φ_PSII_), decreasing the excess energy at PSII and lowering the degree of photoinhibition (F*v*/F*m*) [5]. These findings can be regarded as an economical approach to ameliorate the deficiency of Fe and Zn, which are essential micronutrients for human health [5]. The exposure of crop plants to excess Zn in hydroponics can be regarded as an economical approach to ameliorate the deficiency of Fe and Zn, which are essential micronutrients for human health, and contribute to the achievement of food and nutritional security [5].

Hyperaccumulators that can be used for the phytoremediation of anthropogenically heavy metal-contaminated soils—and also for phytomining, which is the commercial extraction of high-value metals from metal-rich soils—are plant species that actively take up heavy metals, translocate them into the aerial parts, and isolate them into an unhazardous form that is not detrimental to vital enzymes, especially photosynthesis [1,6,7]. Plant endogenous metabolites with a metal chelating ability, called chelators, such as glutathione (GSH), phytochelatins (PCs), and metallothioneins (MTs), are crucial in mediating the adaptation of plants to an excess of heavy metals and supporting the uptake of metals in hyperaccumulator species, providing a tool for increased phytoremediation [7]. These metabolites with metal-chelating ability are summarized in the review of Fedenko et al. [7], with a systemic analysis of phenolics as plant ligands, which are differentiated into subgroups according to the nature of the bioligands based on a modern approach to metallomics. 

Plants have developed various exclusive and effective mechanisms for heavy metal detoxification and tolerance, including the control of metal influx, acceleration of metal efflux, metal chelation and sequestration, metal remobilization, and scavenging of the metal-induced overproduction of reactive oxygen species (ROS) e.g., O_2_^•−^, H_2_O_2_, OH^•^, ^1^O_2_, which are partly reduced or activated forms of atmospheric oxygen (O_2_) [1,8]. Unlike redox-active metals such as Fe and Cu, other metals (e.g., Pb, Cd, Ni, Mn, Al and Zn) cannot generate ROS directly, but only via different indirect mechanisms [1,5]. Metal-induced ROS accumulation is scavenged by enzymatic antioxidants, such as superoxide dismutase (SOD), ascorbate peroxidase (APX), monodehydroascorbate reductase, (MDHAR), dehydroascorbate reductase (DHAR), glutathione reductase (GR), glutathione peroxidase, (GPX), guaicol peroxidase (GOPX), glutathione-S- transferase (GST), and catalase (CAT), as well as non-enzymatic metabolites, such as ascorbic acid, glutathione, a-tocopherol, carotenoids, phenolic compounds, flavonoids, and proline [8]. Although ROS were initially believed to be toxic byproducts of an aerobic metabolism that must be scavenged to avoid oxidative damage to the cell, it is now widely accepted that ROS are used by most organisms as crucial signal transduction molecules that, at basal levels, are essential to sustain life, while intensified ROS production is considered to be beneficial for activating the molecular mechanisms of plant tolerance to metal toxicity [1,8].
